# The Role of Mesenchymal Stem Cell Secretome (Extracellular Microvesicles and Exosomes) in Animals' Musculoskeletal and Neurologic-Related Disorders

**DOI:** 10.1155/2023/8819506

**Published:** 2023-11-07

**Authors:** Alefe Luiz Caliani Carrera, Bruno Watanabe Minto, Patrícia Malard, Hilana dos Santos Sena Brunel

**Affiliations:** ^1^Department of Clinical and Veterinary Surgery, São Paulo State University (UNESP), Av Paulo Donato Castelane s/n, Jaboticabal, São Paulo, Brazil; ^2^Catholic University of Brasilia, Brasília, Federal District, Brazil

## Abstract

The advances in regenerative medicine are very important for the development of medicine and the discovery of stem cells has shown a greater capacity to raise the level of therapeutic quality while their use becomes more accessible, especially in their mesenchymal form. In veterinary medicine, it is not different. The use of those cells, as well as recent advances related to the use of their extracellular vesicles, demonstrates a great opportunity to enhance therapeutic methods and ensure more life quality for patients, which can be in clinical or surgical treatments. Knowing the advances in these modalities and the growing clinical and surgery research and demands for innovations in orthopedic and neurology medicines, this paper aimed to review the literature about the methodologies of use and applications such as the pathways of action and the advances that were postulated for microvesicles and exosomes derived from mesenchymal stem cells in veterinary medicine, especially for musculoskeletal disorders and related injuries.

## 1. Introduction

The initial concepts of stem cells come from the 19^th^ century, when theories were postulated about the tissue cells' short lifespan, as well as their need to regenerate and create new ones to recompose body tissues [1]. Bone marrow multipotent hematopoietic stem cells were discovered more than 60 years ago [2], while mesenchymal cells were mentioned for the first time with this nomenclature only 30 years later when it was discovered that it was impossible to achieve the growth of bone marrow and tissue in another location after the transference of fragments of the original tissues [3]. The popularization of the term stem cells and the recognition by the general population of the theme were obtained, principally, in the middle of the 20^th^ century, when the Dolly sheep's experiment showed the first clinical cloning, using embryonic and also adult cells [4]. The 21st century was marked by the most significant advances in discoveries and developments related to the use of stem cells experimentally, and the beginning of this century was related to the intense clinical trials using this cell lineage [5].

Around the middle of the second decade of this century, the clinical use of stem cells started to occur with more vigor and intensity, secondary to its numerous proofs and assertions regarding the beneficial effects on some pathological processes. Since then, the use has been related to both spheres, in laboratory experimentations or clinical treatments, considering that it is legally available in some countries, especially for mesenchymal stem cells and for veterinary medicine [5]. The use of these cells is not restricted only to human medicine, so it can be applied to veterinary medicine. The application had beneficial effects on a lot of disorders and can also be applied in complementary therapy [6]. Stem cells can be used in different ways, and their origin can be from different tissues and origins, such as autologous, allogeneic, or xenogeneic [4, 5]. Although there are several beneficial effects, the clinical use is still restricted to experimental studies and needs more proof for some administrations, making its commercial use limited in human medicine [6], while in veterinary medicine, the use becomes more popular and evident commercially day by day.

## 2. Stem Cells and Mesenchymal Stem Cells (MSCs)

Stem cells are classified according to their origin, such as embryonic and adult ones. The totipotent ones are those found in the zygote, shortly after fertilization. The embryonic stem cells are those identified in the embryo, in the first days after conception, and may be called pluripotent. The adult cells can be found in the fetus, being differentiated by the tissues they are going to form, such as specificity. They are also called multipotential [7–9]. Multipotent cells are the most identified in clinical routine, being found in specific tissues, such as hematopoietic and mesenchymal stem cells [1, 10]. The hematopoietic cells are those found in the bone marrow stroma and can be differentiated in the blood cells of the different lineages, while the mesenchymal stem cells are found in different body tissues, such as cartilage, adipose tissue, periosteum, and bone marrow, and can be differentiated into cells of the origin tissue and/or signalize for the regeneration of other cells in the same or other tissue [1, 10]. In other words, bone marrow is a reservoir of hematopoietic and mesenchymal stem cells at the same time [11].

**Figure 1:**
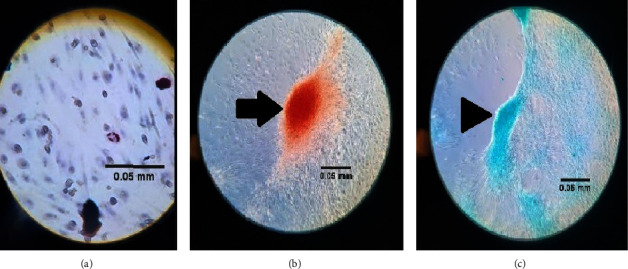
Mesenchymal stem cells' differentiation, through *in vitro* stimulation. After the initial isolation, proliferation, and cultivation, the cell culture was submitted to a culture medium specific for each cellular group cited, resulting in a new tissue formation. Scale bar: 0.05 mm. (a) Adipose tissue, with oil red dye, ×400 microscopic magnification. (b) Bone tissue, with alizarin red dye, ×100 microscopic magnification; arrow indicates the bone tissue formation. (c) Cartilaginous tissue, with Alcian blue dye, ×100 microscopic magnification; arrowhead indicates cartilaginous tissue formation.

Due to ethical and health concerns, totipotent and embryonic (pluripotent) stem cells are not available clinically, being restricted only to experimental environments. Therefore, multipotent cells are the most used, either clinically or in research [10, 12]. Initially known as mesenchymal stem cells, they underwent a reformulation of their nomenclature, because they did not fit some basic inclusion criteria for inclusion in the group of stem cells. After that, its official nomenclature lost the “stem” and started to be called multipotent mesenchymal stromal cells [11, 13]. Following the popular nomenclature, it will be called mesenchymal stem cells (MSCs) in this review. Also, according to the Committee on Tissue and Mesenchymal Stem Cells of the International Society for Cell Therapy, to be classified as a mesenchymal cell by human standards, the following four criteria need to be checked: must be adherent to plastic when maintained under normal culture standards; must be able to differentiate into bone, adipose, and cartilaginous tissues (Figure 1); must express CD73, CD90, and CD105; and must not show cellular markers of c-kit hematopoietic cells, such as CD14, CD11b, CD34, CD19, CD79, and HLA-DR [14].

It is scientifically known that these adult cells differentiate only into cells of the origin tissues or into cells of the tissues to which they are related. However, recent advances showed that through directed stimuli, micromanipulations, and a favorable and specific environment, such cell lineage can adapt and possibly mutate and become a cell of the desired target tissue, even if it is different from the original located in the cell from where it was isolated [10]. So, since they represent a cell group found in various body tissues, such as bone marrow, adipose, cartilage, and bones, in addition to having relatively easy isolation and the possibility to turn into different cell lines, they represent the largest group studied for use in therapies involving stem cells compared to other categories [15].

The most commonly used site for mesenchymal stem cell acquirement is adipose tissue, providing the adipose-derived stem cells (ASCs). This tissue is most commonly used due to its abundance and the reduced side effects caused by its removal. However, factors such as species, breed, age, and adipose tissue deposition rate affect the amount of stem cells available for isolation [16].

## 3. Paracrine Action of MSC with Extracellular Vesicles (EV)

The action of mesenchymal stem cells is related to the stimulation in other cells located at the target tissue, as previously commented [5, 15]. Therefore, through this parameter, numerous recent studies have strived and dedicated to understanding the signaling process of ASC and how it occurs, as well as methods to use them in a more targeted, effective, and safer way [17]. This way it is possible to get only the desired function through the signaling, without the need for direct administration of the cells themselves at the targeted site of action. Studies involving the use of extracellular vesicles from mesenchymal stem cells are recent and had their growth starting in 2012, and these last six years being the most promising in regards to research about the topic [17]. All studies involving this therapeutic modality were performed in animal models, with rats being the most commonly tied to research, while pigs, rabbits, and dogs represented a small group targeted to studies [17].

**Figure 2:**
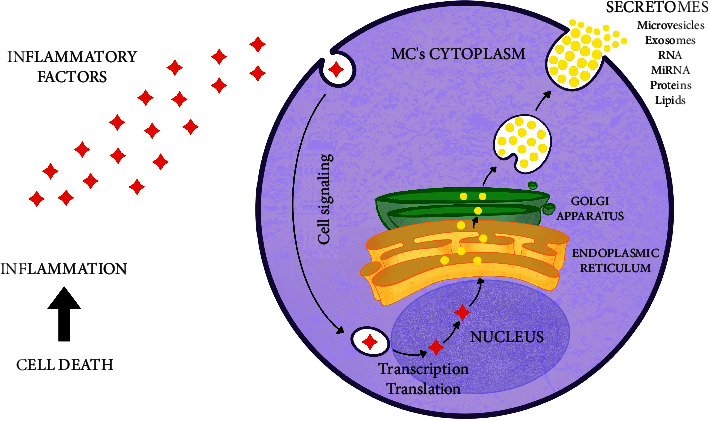
Schematic representation of induction and action of mesenchymal stem cells' secretomes. After cellular damage, a lot of proinflammatory substances are secreted and they act directly at MSC, inducing the transcription and translation of proteins and other factors, which are carried out by vesicles and secreted at the extracellular space, characterizing the secretomes [5, 15, 17]. Figure created with Procreate® software (version 5.3.5, Savage Interactive Pty. Ltd.).

Therefore, it is known that their positive effect on the target tissues is originated by the secreted substances. To get an effect on target tissue cells, it is necessary to establish a mechanism of communication between the MSC and those originating from the local tissue. This signaling is performed in a paracrine way, through the secretion of exosomes and microvesicles, mainly, which form the secretomes (Figure 2). The use of the products of these secretions in the form of exosomes and microvesicles has shown clinical promise because it reduces possible immunogenic side effects caused by the inoculation of cells in the proper form, but with the same desired effect and even potentiated, due to the ability of such compounds to cross biological tissue barriers in a greater manner, secondary to its smaller molecular size [18].

The difference between the secretome molecules is mainly the size of their particles. The microvesicles are larger, about 150 to 1000 *μ*m, while the exosomes are smaller, about 30 to 150 *μ*m. Both structures are surrounded by the cell membrane of the MSCs, containing cytoplasmic material of the originated cell that is delivered into the extracellular environment. Their identification is performed by markers on the cytoplasmic membrane. The vesicles released by the mesenchymal stem cells have innumerable functions as a result of their content. They are responsible for carrying messenger RNA (mRNA) and microRNA (miRNA), as well as proteins and some lipids. The combined effects of these substances can stimulate angiogenesis, apoptosis, reduction of inflammation, and cell proliferation, through the expression of numerous growth factors and proteins related to each desired action [17].

To the correct and desired action, it is necessary to establish communication between the secretome and the cells of the target tissues. This way, for exosomes, it is expected that their intercellular communication occurs mainly in three ways, being interaction of its membrane with the desired cell with contact-dependent signaling and proteins resulting from the cleavage of the exosomes, by the action of proteases, that act as ligands on specific receptors on target cells; and the exosome can fuse with the target cell and transfer its internal material to the cell [19].

On the other hand, microvesicles, unlike exosomes, are calcium-dependent to be formed and can interact with target cells through contact with specific receptors on their membranes. This way, the action is performed by the transfer of specific proteins to the contacted cell or also genetic materials [20, 21]. Microvesicles may act directly on cells or create a favorable environment for their regeneration and replication. They can directly stimulate target cells through membrane receptors and cellular connection, transfer specific receptors to desired cells, transfer functional proteins to those cells, or transfer genetic information through mRNA and miRNA [22].

In the analysis of a compilation of studies involving isolation and laboratory tests of extracellular vesicles of MSC, it was observed that the most used method for isolation of these components was ultracentrifugation or the use of a commercial isolation kit. The differentiation between both categories of the compounds was performed mainly by electron microscopy with morphological characterization, by the Bradford method or BCA, and also by Western blot. Some protein markers can also be used in their classification and identification, such as markers for CD63 and CD9, principally. Bone marrow and adipose tissue are the main tissues from which mesenchymal stem cells can be collected [17].

## 4. MSC and EV as Therapeutic Alternatives in Veterinary Medicine

Even before the birth of the concept and nomenclature of mesenchymal stem cells, their use had already been showing positive results, as shown in the study carried out with bone marrow transplantation for different body tissues. This work analyzed that the bone osteogenic potential was potentiated by bone marrow grafting, through its differentiation into osteoblasts and by the medullar vascular network improvement of this treatment [23]. Since then, a lot of discoveries and developments of techniques have occurred. Animal models of stem cell use are of great importance, both for veterinary medicine itself and for human medicine, whereas several disorders related to dogs resemble human ones and may represent a method for developing new techniques to be applied in human medicine as well [24].

One important point of discussion regarding the use of MSCs concerns from whom they will be taken and what species will give the cells to be used in therapies. The treatment can be performed autologously when the cell is collected and used in the same body. It can also be performed allogeneically when it is collected from an individual to be used in another of the same species. Finally, there is its use xenogeneically, which is characterized by the isolation of the cells from another species to be used [25].

When the subject is the use of stem cells in veterinary medicine, mesenchymal stem cells are the main target of studies and clinical use and tests, since they have no ethical character regarding their extraction, as well as their obtaining and laboratory culture are relatively easy to perform, making this therapy commercially viable [26]. Moreover, its isolation from adipose tissue is the main way to get those cells, due to the abundance of adipose tissue in the animal organism, as well as the easy extraction of it for laboratory cellular culture. Although it presents such advantages, the use of them has been performed more in an experimental way, presenting scarce clinical studies to prove the positive effects of this therapy in natural and nonlaboratory-induced lesions [27]. In addition to the advantages already described, this canine cell lineage has a good therapeutic and morphological response even when submitted to cryopreservation for one year. The osteogenic, myogenic, and adipogenic properties can remain intact, as well as protein expressions. The negative effect of cryopreservation is the reduction of its proliferation potential [28]. These facts open the possibility of keeping isolated cells stored for long periods without major losses in their quality, making this another positive point for their commercial and therapeutic logistics.

In the same way that human MSCs have specific isolation and characterization methods, recent studies have demonstrated ways and patterns to characterize the ones that originated from canine species. Through specific antibody labeling methods, it has been possible to identify that both bone marrow and adipose tissue-derived cells have CD44, CD90, and MHC I while being negative for CD14 and MHC II [29]. These cells derived from adipose tissue, when used therapeutically, can stimulate the production and differentiation of osteocytes, chondrocytes, adipocytes, and tendinocytes and, for this reason, have a wide distribution for use in musculoskeletal disorders [30].

On the other hand, some factors may affect the quality of isolation and action of mesenchymal stem cells. When the analysis was performed for the collection site, comparing the acquisition from adipose and bone marrow tissues about the osteogenic potential, those from bone marrow had 3.5 times greater osteogenic potential compared to those from adipose [29]. Another factor is the age of the animal that will donate the sample of cells. This way, older animals have reduced proliferation power as well as osteogenic effect, while cells from younger animals present a higher number of markers for pluripotential [24].

The use of MSCs in veterinary medicine is mainly based on the treatment of companion and sports animals. The sports animals, principally, are even more affected by this “new” technology. In general, they are mainly used in bone marrow and adipose tissue. Injuries from tendons, muscles, joints, and bones are the main target for the treatment and the goal is to enhance the repair of these tissues through clinical administration [6]. However, there are several systems in which the use of this therapy can bring benefits and improvement of the results [26]. On the other hand, the intravenous administration of MSC may be related to some complication possibilities, as thromboembolism formation is one of them [31]. This way, the therapeutic using of secretome may figure as an important alternative to minimize this complication, with a lot of other advantages related to this therapy [15, 17, 18].

## 5. MSCs and EVs in Veterinary Musculoskeletal Disorders

Musculoskeletal disorders represent one of the most common casuistry in veterinary medicine, both in small and large animals. Therefore, the use of MSC and its secretomes has been widely disseminated in experimental studies [6]. Similar to what occurs in human medicine, extracellular vesicles are also studied and are eligible as more advanced treatment methods in the use of ASCs [32]. They act in the same way, but potentiating the effect, making it more direct and reducing the possibility of side effects, such as hypersensitivity reactions [15]. The use of secretomes can be performed in the form of exosomes or microvesicles [32].

### 5.1. Appendicular Skeletal Disorders

The occurrence of bone injuries, such as fractures, culminates with the abrupt reduction or loss of blood supply. The reestablishment of blood circulation is a primordial factor for the occurrence of fracture repair, being necessary for the nutrients, inherent to ossification, and necessary for delivery to the site of the injury [33]. The blood arrival to the bone involves the plexus of the epiphysis, metaphysis, diaphysis, and cortical and periosteal region, the latter being the most important in terms of the osteogenesis process. In addition, the bone marrow participates in injury repair by supplying osteoblastic precursors, which act in ossification, and chondrocytes precursors, which act in the remodeling of articular cartilage [33].

During healing after the bone crash, there is an initial inflammatory response. This response, among other factors, is responsible for the recruitment of mesenchymal stem cells, which play an important role in the formation of new bone tissue. Then, the MSCs are transformed into chondrocytes, initially forming the cartilaginous callus, which will ensure the initial stability for the necessary neovascularization. The sequence of tissue repair involves the substitution of chondrocytes. They undergo apoptosis. So, osteoblast starts to fill the new bone, brought by the mesenchymal stem cells, and at the same time, the angiogenesis will be stimulated. Finally, there will be osteoclasts' action, which is responsible for remodeling the callus, associated with the maturation of osteoblasts into osteocytes, culminating with tissue regeneration [34].

During these phases of bone regeneration, the participation of endogenous and genetic mediators and stimulators, such as IL6, TNF-*α*, BMP2, IGF, and MMP. During the bone regeneration phase, which involves numerous steps, there is significant involvement of stimulators and mediators of endogenous and genetic origin, such as IL-6, TNF-α, BMP-2, IGF, and MMP [34, 35]. In addition to these molecules, another angiogenic factor can be found, which plays a key role in bone consolidation [34]. Among the mediators that act in the stimulation of bone neovascularization, VEGF (vascular endothelial growth factor), FGF (fibroblast growth factor), and IL8 can be highlighted [33]. There are some bones, such as radius and ulna of small breed dogs that may have a reduced blood supply. The fact of loss of blood delivery can culminate in a reduced healing potential after a crash and surgery [36]. Knowing that, strategies for upgrading this vascularization should take place during the healing process, what could be an important strategy to potentiate the osteoregeneration's effects and to get better results in the surgery treatment. The diminished blood supply to the bone following a traumatic event or surgical procedure can result in a decreased capacity for tissue healing and bone regeneration [36]. Recognizing this, novel approaches to enhance blood flow and vascularization may play a pivotal role in promoting the healing process. Strategies aimed at increasing blood supply could serve as valuable tools to maximize the potential of osteoregeneration and achieve superior outcomes in surgical treatment.

When comparing the ability for osteogenesis in tissues treated with MSCs versus those that received exosomes, it was identified that the use of extracellular vesicles was more efficient in inhibiting oxidative stress, obtained faster DNA repair, as well as obtained inhibition of proteins related to cell senescence and inhibition of cell proliferation [37]. The use of MSCs, for the canine species, has been widespread in cases of atrophic bone nonunion, significant bone loss, and in arthrodesis [38, 39]. For large bone defects in dogs, the use of ASC associated with pedicled omental flap showed superior radiographic and histopathological signs regarding ossification, compared to the group that did not receive ASCs, indicating its upgrade in the osteogenesis process [40].

The use of microvesicles in veterinary orthopedics is recent and still being explored. However, in initial studies, some promising and effective results had been shown and few advances have been reported so far given the recent growth of this technology. Its use in xenogeneic form was proposed with the use of cells extracted from human tissues for use as bone healing enhancers in dogs, showing favorable results [41]. Although it is possible to use ASC and also extracellular vesicles xenogeneically, there are some differences in the cellular secretome according to the species that it is related to. For example, some mechanisms and effects of EV differ between canine and equine species. The equines were found to have increased secretory activity of ASC after magnetic treatment, while the canine ones showed reduced protein secretions [42].

Comparing autologous bone marrow graft, which is routinely used, against ASC's microvesicles for pancarpal arthrodesis surgeries, the groups treated with EV showed better results. In this group, the microvesicle grafting at the surgical site was able to improve the recovery parameters, showing faster radiographic and histological signs of ossification, for the bone marrow group. Moreover, they also showed reduced levels of surgery complications in the other group, decreasing the demand for surgical reintervention. The summing of these results can prove the superior quality of this method and its potential ossification-enhancing power [39]. The use of ASC's exosomes in distraction osteosynthesis for long bones, when bone lengthening was needed, showed positive effects on bone tissue formation, being applied topically in the bone defect. This result can demonstrate another possibility for therapeutic use [43].

ASC-derived microvesicles may show different expressions of their signaling pathways compared to the originating cell. For example, the expression of 32 miRNAs showed differences between the ASC and their microvesicles, being 23 of them more expressed in the EV compared to the mother cell, while nine of them showed the opposite [44]. This difference may be part of the mechanism for such vesicles to have a superior effect on the entire cell during treatments. This RNA group can be classified as noncoding RNA, that is, they are not able to produce signaling proteins by themselves. However, their action pathway is related to the regulation and consequent increase in the expression of mRNAs involved in cellular bone proliferation and maturation signaling, playing a fundamental role in potentiating the genetic response to the stimulus. Furthermore, miRNAs may act through spliceosomes or transport mRNA to the target cell [45]. The main miRNAs involved in EV's action on bone formation are described in Table 1.

The miRNAs of ASC's microvesicles have several pathways to increase angiogenesis in bone repair. This mechanism is very important for the ossification. One of them is through direct activation of two angiogenesis-related genes, Iet-7i-5p and Iet-7f-5p [44]. A study was conducted that observed that the induction of neoangiogenesis represents the main power of improving osteogenesis in extracellular vesicles secreted by ASCs [52]. The study contemplated the use of three groups, one being a control, another using vesicles, and another using vesicles plus vascular endothelial growth factor (VEGF) inhibitor. The difference noted between the groups was significantly related to the formation of vascular tissue and the presence of osteoblasts at the site of the bone defect, being greater in the group that received just the secretome treatment. Furthermore, it was noticed that the formation of bone tissue was directly proportional to the vascular networking developed. This way, the secretome group showed better new bone deposition at the defect. Translating this information into numbers, the group treated with EVs showed a bone tissue forming capacity twice greater than the control group and almost four times more than that compared to the VEGF-inhibitor group [52].

The mechanism involving the stimulation of bone angiogenesis caused by EVs is related to the gene expression regulation, which includes p53, p21, p65, Hippo-YAP, JAK-STAT, *β*-catenin, and PTEN. Of these, the last is the most affected and is considered an oncogenic-control gene. Suppression of its expression may cause procancer effects, with activation of the AKT/mTOR pathway, culminating in angiogenesis, and that is the proposed effect caused by secretomes [53].

As for the cellular and genetic influence caused by EVs on bone healing, some mechanisms can be highlighted. It was observed that the exosomes effectively promoted the expression of *β*-catenin via WNT, an important factor responsible for cell differentiation, providing balance in osteogenic maturation [37]. Another signaling factor associated with the osteogenic power of exosomes is the activation via MAPK (mitosis-activating protein kinase), which also has the effect of stimulating the proliferation of osteoblasts and their maturation in osteocytes, increasing bone tissue [54].

Also, genetic potentials associated with osteoblastic stimulation for bone regeneration are postulated. Some of the mechanisms are through the stimulation of extracellular vesicles to increase the expression of mRNA derived from runt-related transcription factor 2 (RUNX2), osteocalcin (OC), and osterix (OSX) genes, which will promote the transcription of proteins that act in osteoblast differentiation and maturation of bone tissue [55].

The exosomes, through *in vivo* evaluations, demonstrated an increase in cell proliferation, in alkaline-phosphatase (ALP) activity, and in the mRNA expression from osteoblast-related genes, such as OPN, RUNX2, and COL1. *In vivo*, the results were consistent with the new bone formation induction, as well as increased bone vascularization. Another fact demonstrated was that the response was directly proportional to the concentration of exosomes in the treatment used in the study. When the concentration of exosomes was increased, a more satisfactory response was observed to the parameters cited before [56].

Thus, the effects of these substances are positively related to the potentiation of bone repair through an upgrade of tissue formation. However, better methods of their application are still being investigated to result in a satisfactory use. The intravenous administration of exosomes may not present a desirable response in promoting osteoinduction. However, this effect can be achieved more satisfactorily when exosome complexes associated with aptamers are formed, which, after intravenous administration, can promote the targeting of bone tissue of the vesicles, and have satisfactory results in enhancing bone healing [57]. In addition to systemic administration, topical use can be performed in a variety of ways.

The use of exosomes associated with different types of hydrogels locally in osteosynthesis also showed positive results. Its reported effects were increased proliferation and differentiation of osteoblasts progenitor cells, increased BMP2 (bone morphogenetic protein 2), greater vascular density, and consequently, greater deposition of bone tissue compared to the other groups evaluated [58]. Similarly, its use manipulated together with tricalcium phosphate-based bone cement is one of how it can be used. The bone cement will promote the coverage of the bone defect as well as allow the slow release of exosomes and their components to act on the target cells. This method of use has revealed a potentiating effect of osteoregeneration when compared to the use of cement alone. The activity of this compound is also related to genetic signaling of the PI3K/AKT/mTOR pathway, increasing the osteogenic response [59]. Also, its topical administration can be performed in beads of bioabsorbable bone cement of hydroxyapatite and/or tricalcium phosphate. Through a study, it was identified that the slow release of EVs promoted by the bone cement was favorable to tissue healing and for promoting the improvement of the osteogenic potential [41].

Demineralized bone matrix, which is used routinely as an allograft, can also be treated with microvesicles, which is another method of manipulation of bone graft. This method showed advanced potential in ossification, compared with the nontreated bones. The use of EVs demonstrated a greater bone tissue formation effect, with increased collagen fibers, osteocalcin, and osteopontin [60]. Also, the hypoxia treatment of ASC showed favorable effects regarding their secretomes in bone metabolism. MSCs under hypoxia conditions have an increased secretion response for EV. Therefore, the utilization of hypoxemic MSC's secretomes represented a greater osteogenic power compared to those conditioned under normal oxygen concentrations [51]. The mechanism involved in the upgrade in osteogenic potential in hypoxia EV is related to the activation of the HIF-1*α* (hypoxia-induced factor 1) gene, which may lead to the overexpression of miRNA-126 [51].

The use of dexamethasone in mesenchymal cell culture can affect the secretion of extracellular vesicles and positively affect their bone production capacity. The mechanism of action was shown to refer to increased proliferation of MC3T3 (osteoblastic precursor cells) and increased expression of RUNX2, alkaline-phosphatase, and osteopontin, all mechanisms culminating with greater bone tissue production [54]. In contrast, the action of MSC's microvesicles from patients who underwent steroidal anti-inflammatory therapy showed reduced effects. Their use in promoting osteogenesis and osteoinduction revealed lower potential than patients who did not receive the drug. So, it was concluded that the systemic corticosteroid therapy may reduce the beneficial effects of EV [61]. On the other hand, other types of cellular treatment can modify the results of EV's isolation and effectiveness. The exposure to ASC to magnetism revealed positive results regarding the production of EVs. In this context, the production of secretomes was higher in cultures exposed to magnetic waves, as well as microvesicles and exosomes presented a greater presence of growth factors compared with those of unexposed group. This may be a form of cell culture to further potentiate the therapeutic capacity of secretomes [42].

### 5.2. Joint Diseases

The frequency of joint disorders in the clinical routine of small animals is elevated and arthritis diagnoses are frequent and from diverse origins. Joint inflammations configure the occurrence of osteoarthritis. In general terms, the mechanism that leads to joint damage is similar, independent of the disease origin. Initially, the inflammation occurs with osteophyte formation, joint remodeling, and degeneration of the cartilage and subchondral bone, according to the progression of the disease. Through inflammation, there will be increased vascularization of subchondral bone. This way, there will be an increase in inflammatory cell circulation and a sequential increase in joint resorption by the action of osteoclasts. The action of inflammatory cells will culminate with the release of cytokines, interleukins, and other inflammatory mediators such as MMP, TNF-*α*, and IFN-*γ*, for example. As a result, osteoblastic activation, hypertrophy, apoptosis of chondrocytes, and deposition of collagen fibers occur, culminating in macroscopic changes of joint erosion, bone sclerosis, and periarticular fibrosis. Systemic factors may be tied to worsening the articular injury and consequently delay clinical and histological recovery [62–64].

When it comes to cartilaginous lesions, the absence of vascularization represents an important delay in their healing, and this fact is very important because the healing process is dependent on regeneration, which needs more blood nutrition to take place. In this context, alternative therapies to accelerate the healing process are important and MSC, principally ASC, and its extracellular vesicles are among the techniques with high potential for therapeutic application [65]. The use of mesenchymal cell secretomes from adipose tissue is shown as an effective and potential therapy for osteoarthritis cases. Its effects may be related to the stimulation of chondrocyte replication and stimulation of articular stem cell differentiation [66].

**Figure 3:**
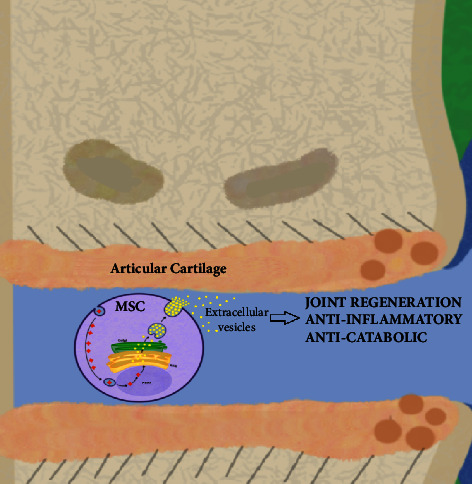
Schematic representation of intraarticular action of mesenchymal stem cells' secretome. After intraarticular administration or delivery from the bloodstream, a lot of substances inherent to the extracellular vesicles are secreted and they act in the articular cartilage and environment, resulting in anti-inflammatory and anticatabolic effects, in addition to the stimulation of joint regeneration and resurfacing [67–75]. Figure created with Procreate® software (version 5.3.5, Savage Interactive Pty Ltd.).

Its use shows positive results in reducing the clinical signs of patients affected by chronic inflammation of the joints, even when performed with cells of allogenic origin, thereby reducing the levels of clinical pain to almost the half compared to the control group [67]. Similarly, extracellular vesicles are efficient in different ways. One of them is the regenerative potential. Another is also the positive response in reducing joint inflammation in clinically-induced osteoarthritis patients, especially when used intraarticularly [68] (Figure 3). Its action pathway is also shown to be beneficial in severe joint defects in animal models, where intraarticular use in three-weekly administration, associated with hyaluronic acid, was shown to be effective in both cartilage and subchondral bone repair [69]. When used in the treatment of joint defects, microvesicles also showed very positive results compared to the placebo-treated group of dogs. Through surgically created defects in the femoral condyle, the groups that received intraarticular treatment with ASC-derived microvesicles showed signs of cartilaginous and subchondral bone regenerations, unlike the placebo group, which showed joint degeneration and deposition of fibrous tissue, forming fibrocartilage. In this study, it was observed that regenerative therapy caused satisfactory functional and morphological recovery [70]. Microvesicles extracted from mesenchymal cells of articular cartilage also showed positive ability when used in the treatment of osteoarthritis. Topical administration of these microvesicles intraarticular demonstrated an inducing effect of chondrocyte regeneration, reducing apoptosis via FOX class O (FOXO) proteins, and stimulating the action of M2 macrophages in regulating inflammatory response through increased IL-10 [71].

In the same manner as the mechanisms of action, there are a lot of ways related to genetic influence and protein modulators to culminate with the benefits results. The reduction of joint inflammation response occurs through the mechanisms of decreased production of proinflammatory cytokines, reduced expression of MMP13, inhibition of macrophage activation, and intraarticular nitric oxide release, thus reducing the oxidative stress of the joint [66]. Its pathways further include stimulation of tendon progenitor cells (TSPC) proliferation and differentiation, reduction in catabolic and inflammatory markers, increasing chondrocyte proliferation through genetic stimulation of GIT1, and increasing chondrogenic markers in chondrocyte precursor cells [72–75].

The induction of miRNA overexpression in the MSC's secretomes is an analytical method to evaluate their actions and potential genetic modifications to promote their expected activity. Laboratorially, the induction of increased expression of a specific miRNA was performed to evaluate its potential effect in the treatment of osteoarthritis define the pathways that each other can act, and elucidate any of them's function. The functional characterization of individual miRNAs was conducted through controlled iatrogenic overexpression. This methodology enabled the evaluation of their therapeutic potential in the context of osteoarthritis, elucidation of their associated signaling pathways, and the comprehensive understanding of their mechanistic roles [66]. The miRNA-381 promoted chondrogenesis by the stimulation of the TAOK1 gene, increasing the expression of threonine-protein kinase-1 [76]. The miRNA-92a-3p increased chondrocyte proliferation and migration by suppressing the protein expression of the WNT5A gene [77]. The miRNA-95-5p inhibited HDAC2/8 gene expression and promoted chondrogenic cell differentiation of MSCs and cartilage matrix production [78]. There was also the participation of miRNA-320c, which increased chondrogenic proliferation and differentiation of MSCs into chondrocytes [79], as well as inhibited MMP13 and interleukin-1*β* production [80].

In another study, besides the reduction of IL-1*β* expression, an increase in PRDX6 gene expression was identified, forming high levels of peroxiredoxin-6, reducing the levels of free radicals and joint oxidative stress, this being one of the mechanisms of action to reduce the articular damage and oxidation. In addition, microvesicles increased the gene expression of LC3B, a marker of the autophagy process [81]. ASC's microvesicles and exosomes also revealed antioxidant effects in the joint space. The reduction of oxidation can decrease the senescence of joint cells and their apoptosis, in the same way that reduces inflammatory mediators, such as IL-6 and prostaglandin E2 [82]. The high expression levels of sphingosine-1-phosphate (S1P), resulting in the overproduction of sphingosine kinase-1 (SphK1), is another way of action related to microvesicles in the joint through genetic involvement. S1P is responsible for the activation of S1PR1 receptors on the membrane of chondrocytes. This receptor may act to induce cellular replication and articular repair. This pathway can be activated by microvesicles that are positive for CD44 [83].

According to the method of administration, the secretomes may be used in dogs intraarticularly after pharmaceutical manipulation, being prepared as a lyophilized powder. This product, after manipulation, is administered in the joint space, showing safety, absence of side effects, and good results of therapeutic parameters [84]. Knowing that this pharmaceutical compound is promising and able to be commercially more acceptable, it is easy to handle and safe to use in clinical-hospital environments.

### 5.3. Ligaments and Tendons Diseases

The therapeutic approach for tendon disorders is also another appliance to MSC secretome therapies. The topical administration of exosomes was able to upgrade the fibrocartilaginous tissue formation at the site of the lesion, decreasing the apoptosis and increasing the cellular proliferation through the higher levels of M2 macrophages chemotaxis to the site of injury, getting better recovery to the tendon's biomechanical properties. The MSC secretome therapies are also applicable for the treatment of injuries involving tendons and ligaments. In the case of tendon tissue, the local administration of exosomes demonstrated an ability to enhance local fibrocartilaginous tissue production, reduce apoptosis, and increase cellular proliferation. In addition, there was an observed increase in the recruitment of M2 macrophages to the injury site, resulting in improved restoration of the biomechanical properties of the tendon tissue [85]. For ligament injuries, microvesicles also show better properties and results. Through an *in vitro* study, it was observed that secretomes were able to promote local mesenchymal stem cell proliferation and had antiapoptotic proprieties. Thus, when it was used *in vivo*, the therapeutic approach could increase the angiogenesis and the tissue elasticity, as well as promote the fulfillment of the site of injury [86].

## 6. MSCs and EVs in the Treatment of Axial Skeletal, Central, and Peripheral Nervous System Disorders

In appendicular skeletal disorders, the clinical care of spinal injuries represents a large casuistry in the clinical routine of small animal practice, involving bone and spinal cord disorders, intervertebral disk diseases, and peripheral nerves [87]. Likewise, the encephalic involvement is one of very clinical importance too. In this sense, MSC and its EVs are also applicable, with anti-inflammatory effects on the central nervous system and stimulating neuronal tissue regeneration. The involvement of encephalic tissues also holds significant clinical importance. In this context, both MSCs and EVs are viable for therapeutic approaches, dependent on the condition, yielding anti-inflammatory effects and promoting neuronal regeneration in the central nervous system[88]. In addition, they have neuroprotective ability. After tissue damage in the central nervous system, the use of mesenchymal stem cells was able to induce the production of some substances that had the power to stimulate tissue repair through regeneration. In the same way, they were able to stimulate the action of local stem cells of the nervous tissue to repair the damage. The sum of both actions showed its satisfactory and promising effect on injuries related to nervous tissues [89]. The neuroprotective and regenerative power may be related to the genetic proprieties that MSCs carry on. In their genetic compound, sequences related to the formations of neurons and glial cells were found. So, knowing that their effect may be promising through the regenerative and stimulation power to nervous system tissue, which is not restricted only to neurons, but to the whole cell range involved in the functioning of this tissue [90], the substances secreted by these cells can reduce neuroinflammation and myelin degeneration as well as promote the myelination effect in neurons [91].

Although they carry on nervous tissue sequences in their genetic material, the differentiation of those cells into neurons is difficult to occur when the test is performed in laboratory stimulation. The occurrence was partially identified, through the expression of neurogenin-1 [92]. Therefore, it is understood that the MSC effect is more related to the stimulation of tissue regeneration and repair than to its mutation into neuron cells themselves. Thus, the nervous system results of clinical applications of MSCs are not restricted only to the central compounds and also show positive results when used in the peripheral nervous system. In this case, one of the actions of ASCs is the stimulation of peripheral neuroregeneration through paracrine and exosome secretions, in addition to mutation potential in glial cells, mainly in Schwan ones, which represents an important peripheral neuro cicatricial role [93–95].

Mesenchymal stem cells showed beneficial effects in the regeneration of nerve function after acute spinal cord injury in dogs [96], as well as for dogs that were affected by intervertebral disk disease (IDD) in the thoracolumbar spine, without deep nociception. In this last case, the use of ASC associated with surgical decompression showed better results in patient recovery. These animals presented, on average, half of the time needed to recover deep pain perception and less time to achieve complete neurological function recovery compared to those who did not receive the cellular treatment. Also, complete recovery of neurological clinical function was achieved in more than 55% of patients who received cell therapy [97] and results demonstrate a high therapeutic potential given the enormous casuistry of canine patients affected by intervertebral disk disease with severe neurological sequelae. Furthermore, ASC may be applicable in the treatment of chronic intervertebral disk injuries, without surgical treatment. In a study conducted on patients with IDD, ASC culture was administered intravenously every 30 days, four times, summing four months of treatment, associated with physical therapy rehabilitation. There was an improvement in locomotion and joint movements, as well as an improvement in urinary sphincter control, reducing the incontinence in half of the patients [98]. These results have a great importance through the secretion of extracellular vesicles, which can also be used alone in the treatment of these disorders.

The extracellular vesicles can be used through intrathecal administration for acute spinal cord damage. Their actions are related to anti-inflammatory mechanisms, by reducing the expression of IL-18, IL-1*β*, and other cytokines, and also showed a reduction of neuronal death and an increase in the regeneration of them, with consequent stimulation of the return of movement [99]. Another mechanism involved in the microvesicle treatment efficacy for acute spinal cord injuries involves the expression of miRNA-381, which has an inhibitory effect on the BRD4 and WNT5A gene pathways [100]. Microvesicles can be applied as a therapeutic alternative for central nervous tissue lesions, acting in the encephalic region to control damage caused by excitotoxicity injury. Through laboratory findings, it was identified that they also act through the PI3K/AKT pathway, reducing caspase-3 cleavage and Bax expression, acting to reduce toxicity injury caused in neurons [101]. On the other hand, vesicles demonstrated satisfactory anti-inflammatory effects for glial cells through a laboratory model using BV2 cells, by reducing IL-1*β* production [102].

The action of MSCs and their secretome is also directly applicable to the intervertebral disk. The degenerative process is common and can be a possible target for exosomes, which were able to induce the proliferation of nucleus pulposus' cells and increase extracellular matrix production [103] after injection inside the disk. The mechanism of action is related to the increased expression of miRNA-21 caused by exosomes. This RNA was able to reduce cell apoptosis mediated by TNF-*α*, in addition to promoting a negative modulation of PTEN expression, resulting in its inhibition, and activating the PI3K/AKT pathway in the apoptotic nucleus pulposus [104]. In the same way, the action is satisfactory to peripheral nerves. The use of EV promoted histomorphometric and functional nerve repair, increasing the expression of GAP-43, which acts as a marker of axon regeneration [105].

## 7. MSCs and Their Secretome in Soft Tissue's Regeneration

Orthopedic injuries occur, a lot of times, concomitantly with soft tissue damage, such as skin, subcutaneous tissue, fascia, and muscles, for example. The methods to increase soft tissue healing are also of great importance and arouse the concern of orthopedic surgeons in the management of open fractures. Therefore, it is important for surgeons to know how these techniques favor tissue recovery. In this context, extracellular vesicles have several healing-enhancing effects, which are also linked to the bone repair, especially neovascularization, with positive effects even when performed allogeneically [106]. The administration of a solution containing ASC-derived extracellular vesicles subcutaneously and in the perilesional region, in dogs with skin wounds, it was observed effects of increased synthesis of collagen and vascular network. The perilesional subcutaneous administration of extracellular vesicles derived from ASC, in dogs undergoing skin wounds, resulted in increased collagen synthesis and improved vascular networking. Related to the vascular tissue, in addition to promoting new-vessel formation, it was also able to promote in a faster way their development and maturation, making the whole healing process and wound retraction much faster [107].

The other aspect of EVs is the microvesicles. The use of them in the treatment of skin injuries in dogs showed more satisfactory effects compared to conventional treatment, even when allogeneic microvesicles were used and administered directly at the focus of the wound. Through histological evaluation of treated tissues, it was observed that EV-treated skin showed a greater formation of the vascular network, as well as greater area of interaction between the dermis and epidermis and a greater definition of the papillary layer, which plays an important role in nourishing the epidermis [108]. The effect of increased vascular network seems to be related to the carriage of a miRNA by microvesicles. According to recent studies, miRNA-31 has the inhibitory effect of the HIF-1 gene, an antineoangiogenic gene, and this may be its main way of action to generate such an effect [109]. Also, the use of exosomes derived from adipose, bone marrow, and umbilical cord mesenchymal stem cells represented significant expression of vascular endothelial growth factor A (VEGF-A), fibroblast growth factor 2 (FGF-2), hepatocyte growth factor (HGF), and platelet-derived growth factor BB (PDGF-BB), besides inducing migration and proliferation of keratinocytes and fibroblasts in the soft tissues. In these last two effects, the response is dose-dependent, which means that the higher the dose, the greater the response to migration and proliferation [110].

## 8. Final Considerations

Considering the constant innovations and relevant discoveries regarding the use of stem cell therapy, new ways of using them have been routinely presented to the medical community. Studies of new approaches are described in Table 2. As a literature review, this article aims to clarify some information about this new cellular therapy that is growing in the field of veterinary medicine, using expressive results available in the scientific literature. Despite this, the reduced number of studies for specific diseases figures as a limitation for a deep decision-making about the correct methods for administration and expected results of the therapies, which is expected for therapies that are emerging for clinical usage. It is expected that by shining the light on this cellular resource and review, new studies may occur and originate new reviews in the future with more answers for specific disorders related to companion animals.

The secretomes have shown numerous advantages in laboratory studies but their clinical use still requires parsimony. The main limitation of its use is related to the ethical and legal parameters for production and clinical safety for commercial distribution, principally related to the totipotent and pluripotent stem cells [18]. For their safe use, it is expected that there will be new studies to clarify some doubt, such as methods to produce them on a large scale and quantify and characterize them and their contents quickly and accurately, which is necessary for commercial production. Another necessary analysis is about their pharmacokinetics and pharmacodynamics, the ways of migration to target tissues, as well as safe methods of administration and determination of safe and toxic doses and the possible side effects to its application [18]. Given the more positive effects of using extracellular vesicles in complementary therapies, this is the focus of the study to be addressed by researchers for future studies involving stem cells. In the field of veterinary and human medicine, the aspects related to orthopedic and neurology therapies are the ones that most need studies and innovations, laboratory and clinical proofs and validations to be possible the commercial distribution of the secretomes to improve patients' welfare and better methods to include this alternative therapy in the clinical routine of veterinary hospitals. Considering the favorable outcomes associated with the utilization of extracellular vesicles in adjunctive therapies, the focus of future research endeavors in the realm of stem cells lies in this direction. Within the domains of veterinary and human medicine, orthopedic and neurological therapies represent areas that demand heightened scrutiny, necessitating rigorous laboratory and clinical investigations for validation. These studies are integral to enabling the commercial dissemination of secretomes with the goal of enhancing patient welfare and establishing effective procedures for the integration of this alternative therapy into the clinical routines of veterinary hospitals.

## 9. Conclusion

Extracellular vesicles derived from mesenchymal stem cells are a novel method for alternative therapies in the clinical routine, especially for veterinary medicine, and the adipose-derived figure is the major one, due to the easier isolation. To the common casuistry, the disorders related to the musculoskeletal system may figure as an important target for therapies but still remain poorly investigated for specific disorders in companion animals. Further studies are encouraged to deeply understand the future directions of this therapy.

## Figures and Tables

**Table 1:** Action of mesenchymal stem cells' extracellular vesicles (EV) through miRNA's expression and production on bone formation.

EV's action	miRNA	miRNA's action	Reference
Reduce expression	miRNA-144, miRNA-31, and miRNA-221	Inhibitors of osteogenesis	[46]
Increase expression	miRNA-21	Improvement in bone production	[46]
Increase expression	miRNA-146a-5p, miRNA-503-5p, miRNA-483-3p, and miRNA-129-5p	Related positively to new bone formation	[47]
Inhibition of action	miRNA-32-5p, miRNA-133-3p, and miRNA-204-5p	Activation of PI3K/AKT and MAP-kinase pathway, reducing osteogenesis	[47]
Production and secretion	miRNA-375	Activation of the IGFBP3 gene, responsible for increasing osteoblast proliferation	[45]
Production and secretion	miRNA-148-a	WNT5A/Ror2 pathway, reducing adipogenic differentiation, and increasing osteogenic potential	[48]
Production and secretion	miRNA-877	Increased expression of BMP2, which increases angiogenesis and bone deposition	[49]
Overexpression	miRNA-126	Induces osteoregeneration and VEGF's expression	[50]
			[51]

**Table 2:** Description of new studies involving extracellular vesicles of mesenchymal stem cells related to the clinical routine of veterinary clinicians.

Species	Disease	Type of treatment	Response	Conclusion	Reference
Dog and human	Autoimmune diseases	Extracellular vesicles	Anti-inflammatory and immunosuppressive effects	Positive effect	[15]
Dog	Inflammatory bowel disease	Extracellular vesicles	Decrease of TNF-*α*, IL-1*β*, IFN-*γ*, IL-6, and IL-10	Positive effect	[111]
Dog	Chemotherapy-induced dilated cardiomyopathy	Extracellular vesicles	Improve cardiac function and reduce myocardial apoptosis and fibrosis	Positive effect	[112]
Dog	Chemotherapy	Microvesicles	Release-control of the drug	Positive effect	[113]
Dog	Atopic dermatitis	Extracellular vesicles	Reduce the expression of cytokines and interleukins; activate the JAK/STAT pathway	Positive effect	[114]

## Data Availability

The data used to support this study are available within this study. All articles cited and information collected in this review were searched in academic databases.
